# Clinical Characteristics, Treatment Patterns, and Outcomes of Chinese Patients With Alopecia Areata: A Retrospective Observational Study

**DOI:** 10.1111/jocd.71045

**Published:** 2026-07-14

**Authors:** Youyu Sheng, Zhibing Qiu, Jun Zhao, Lijuan Zhou, Ying Miao, Bilian Zhao, Jinnan Li, Guanshen Dou, Wenyu Wu, Qinping Yang

**Affiliations:** ^1^ Department of Dermatology Huashan Hospital, Fudan University Shanghai China; ^2^ Department of Digestive Disease Huashan Hospital, Fudan University Shanghai China; ^3^ Lilly China Drug Development and Medical Affairs Center Shanghai China

**Keywords:** alopecia areata, disease course, quality of life, retrospective study, treatment patterns

## Abstract

**Background:**

Alopecia areata (AA) is an inflammatory, nonscarring hair loss disorder with significant variations in the clinical presentation and unpredictable nature of the disease. Although AA is common in clinical dermatology, knowledge gaps remain regarding the clinical characteristics and impact on patients' psychological well‐being and overall quality of life.

**Aims:**

This study aims to evaluate the clinical characteristics, quality of life, treatment patterns, and long‐term prognostic outcomes in Chinese patients with AA.

**Methods:**

We conducted a retrospective observational study at Huashan Hospital affiliated to Fudan University, enrolling patients with AA treated in the outpatient clinic between 2018 and 2024. Data on demographic features, clinical manifestations, quality of life, treatment patterns, and disease progression were collected. Descriptive statistical analyses were conducted to summarize the variables of the overall sample. Differences in the above variables were analyzed between mild‐to‐moderate and severe AA.

**Results:**

A total of 70 patients with AA were enrolled in this study. The median age at the time of visit was 37.0 years (interquartile range [IQR], 30.0–45.0 years). By Severity of Alopecia Tool (SALT) score, 29 patients were classified as mild‐to‐moderate AA, whereas 41 patients were categorized as severe AA. The patients' quality of life was significantly impaired, as evidenced by a mean Dermatology Life Quality Index (DLQI) score of 16.5 (standard deviation [SD], 6.7). For treatment patterns, the combination of immunomodulatory drugs and nutritional supplements was the most common option. For the disease course, 72.9% recovered with recurrence.

**Conclusion:**

In conclusion, this retrospective study characterized the clinical features, quality of life, treatment patterns, and long‐term prognosis of Chinese AA patients. Current therapies fail to achieve optimal complete hair regrowth and recurrence reduction, leaving significant unmet needs in AA management.

## 1 Introduction

Alopecia areata (AA) is an autoimmune condition characterized by patchy or extensive nonscarring hair loss on the scalp or any hair‐bearing surface [1]. The prevalence of AA is estimated at 0.1%–0.2%, with a lifetime risk of 1.7%–2.1% in the general population worldwide [2]. Clinically, AA manifests in diverse forms, including patchy, ophiasis, sisaipho, diffuse, alopecia totalis (AT), and alopecia universalis (AU) [3]. The disease exhibits no sex predilection and demonstrates a broad age range of onset, affecting individuals across all decades of life [4]. The clinical course of AA is unpredictable and varies greatly among individuals. Most patients with AA experience hair regrowth within a few years, but sudden recurrence can occur at any given time [5, 6].

AA commonly exhibits comorbidity with atopic predispositions and a spectrum of autoimmune disorders, such as thyroid disease and vitiligo [7]. Beyond its physical manifestations, AA has far‐reaching psychosocial implications. Recent evidence has demonstrated an association between AA and psychiatric comorbidities, including depression and anxiety, and considerable impacts on patients' health‐related quality of life [8]. Patients with AA frequently encounter compromised self‐perception, interpersonal relationship strain, and disruptions to daily activities, even in those with mild‐to‐moderate AA [9]. Despite ongoing research, the exact cause and precise etiology of AA remain elusive, complicating treatment exploration and development. There are several treatment options for AA with varying efficacy and safety, but they lack curative effectiveness [10]. An increasing amount of evidence indicates that solely using the Severity of Alopecia Tool (SALT) score is insufficient to comprehensively evaluate the overall condition of patients, including the disease duration, the response to treatments, and the psychological and social impacts on the patients [11]. A certain proportion of patients with AA exhibit refractory disease manifestations or experience recurrence after initial treatment [12, 13], underscoring the need for improved therapeutic strategies.

Although AA is a common problem in clinical dermatology, there remains a paucity of objective data concerning the clinical characteristics, treatment patterns, and long‐term prognoses of affected patients [14, 15]. The aim of the current study was to describe the clinical characteristics, quality of life, treatment patterns, and long‐term prognostic outcomes in Chinese patients with AA.

## 2 Methods

### 2.1 Study Design

A retrospective single‐center study was performed, including AA patients treated and regularly followed up for at least one year at the Department of Dermatology, Huashan Hospital affiliated to Fudan University from August 2018 to August 2024 under IRB‐approved protocols (2021–11 042). Each patient who voluntarily agreed to participate provided written informed consent.

### 2.2 Data Collection

Patient demographic data were collected, encompassing age at the time of the visit, sex, occupational status, smoking and alcohol consumption habits, dietary habits, outdoor activities, physical exercises, sleep patterns, and family history of AA. Clinical information, including age at onset, age at diagnosis, clinical subtype and stage of AA, Severity of Alopecia Tool (SALT) score, eyebrow and eyelash involvement, Dermatology Life Quality Index (DLQI), comorbidities, treatment process, and the outcome of AA, was recorded [16]. A SALT score of 0 was regarded as indicative of complete recovery. The entire course of AA of each patient from the first onset to the most recent visit, including recurrence frequency, recurrence intervals, and duration of each disease episode, was documented. The recurrence rate was defined as the proportion of patients with recurrence, and the cumulative recurrence rate was calculated at various time points.

### 2.3 Statistical Analysis

Continuous variables with a normal distribution were reported as mean and standard deviation (SD), while median and interquartile range (IQR) were presented for non‐normal continuous variables. Categorical variables were summarized as frequencies and percentages. Results were reported for the total samples and stratified into mild‐to‐moderate AA and severe AA groups. Differences between mild‐to‐moderate AA and severe AA groups were tested using the Wilcoxon rank‐sum test or independent *t*‐test for continuous variables, and the Fisher's exact test or chi‐square test for categorical variables. All statistical analyses were performed using SAS software (version 9.4), with a statistical 2‐sided significance level defined as *p* < 0.05.

## 3 Results

### 3.1 Demographic Characteristics

Seventy patients with AA were enrolled in the present study, including 29 patients with mild‐to‐moderate AA and 41 patients with severe AA. Table 1 presents the demographic characteristics of patients with AA at the time of their initial visit at Huashan Hospital. The median age of all patients at visit was 37.0 years (IQR, 30.0–45.0). Most patients were female (85.7%). A family history of AA was reported in 27.1% patients. There were no significant differences in the above demographic characteristics between patients with mild‐to‐moderate AA and those with severe AA.

**TABLE 1:** Demographic characteristics of patients with alopecia areata according to disease severity.

Characteristics	Total (*N* = 70)	Patients with mild‐to‐moderate AA (*N* = 29)	Patients with severe AA (*N* = 41)	*p*‐value
Age at visit, median (IQR), years	37.0 (30.0–45.0)	39.0 (30.0–44.0)	37.0 (29.0–45.0)	0.555
Sex (%)				0.178
Males	14.3	6.9	19.5	
Females	85.7	93.1	80.5	
Occupational status (%)				0.359
Employed	92.9	93.1	92.7	
Retired	5.7	6.9	4.9	
Unemployed	1.4	0	2.4	
Smoking status (%)^a^				0.726
No	87.1	89.7	85.4	
Yes	12.9	10.3	14.6	
Alcohol status (%)^b^				0.566
No	95.7	93.1	97.6	
Yes	4.3	6.9	2.4	
Physical exercise (%)				0.707
Never	67.2	62.1	70.7	
1–2 times per week	27.1	31.0	24.4	
3–5 times per week	5.7	6.9	4.9	
Family history of AA (%)				0.283
No	72.9	65.5	78.0	
Yes	27.1	34.5	22.0	

*Note:* Data were presented as median (interquartile range) for continuous variables and as percentages for categorical variables. The Wilcoxon rank‐sum test was used for continuous variables and Fisher's exact test for categorical variables.

Abbreviations: AA: alopecia areata; IQR: interquartile range.

^a^ Smoking was defined as current or former tobacco use.

^b^ Alcohol was defined as current or former alcohol consumption.

### 3.2 Clinical Characteristics

Table 2 summarizes the clinical characteristics of the 70 patients with AA. The median age at onset was 30.4 years (IQR, 18.7–40.1), and the median age at diagnosis was 34.4 years (IQR, 26.7–41.0). In addition, the mean disease duration was 8.4 years (SD, 7.0). The most prevalent clinical subtype of AA was multiple patches, accounting for 62.9% of cases. Furthermore, alopecia totalis (AT) and alopecia universalis (AU) were observed in 7.1% and 2.9% of AA patients, respectively.

**TABLE 2:** Clinical characteristics of patients with alopecia areata according to disease severity.

Characteristics	Total (*N* = 70)	Patients with mild‐to‐moderate AA (*N* = 29)	Patients with severe AA (*N* = 41)	*p*‐value
Age at onset, median (IQR), years	30.4 (18.7–40.1)	30.9 (15.3–38.3)	30.3 (20.4–40.1)	0.986
Age at diagnosis, median (IQR), years	34.4 (26.7–41.0)	36.6 (27.4–41.0)	32.4 (26.3–40.9)	0.456
Disease duration, mean (SD), years	8.4 (7.0)	9.6 (8.9)	7.6 (5.2)	0.277
Clinical subtype (%)*				< 0.001
Single patchy	10.0	24.1	0.0	
Multiple patches	62.9	72.5	56.1	
Diffuse AA	4.3	3.4	4.9	
Alopecia ophiasis	11.4	0.0	19.5	
Alopecia totalis/alopecia universalis	11.4	0.0	19.5	
Stage of AA (%)*				0.029
Progressive stage	71.5	82.8	63.4	
Stable stage	27.1	13.8	36.6	
Recovery stage	1.4	3.4	0.0	
Comorbidities associated with AA (%)				0.321
Yes	24.3	20.7	26.8	
Allergic rhinitis	11.4	13.8	9.8	
Hashimoto's thyroid	7.1	0.0	12.2	
Asthma	2.9	3.4	2.4	
Chronic urticaria	2.9	3.4	2.4	
Vitiligo	2.9	0.0	4.9	
Atopic dermatitis	1.4	0.0	2.4	
Diabetes	1.4	3.4	0.0	
Sjögren's syndrome	1.4	3.4	0.0	
SALT score, mean (SD)*	52.6 (38.6)	9.2 (8.0)	83.3 (14.2)	< 0.001
Body hair loss (%)*				0.015
No body hair loss	50.0	69.0	36.6	
Partial body hair loss	50.0	31.0	63.4	
Eyebrow ClinRO score, mean (SD)*	1.0 (1.2)	0.5 (1.0)	1.3 (1.2)	0.003
Eyelash ClinRO score, mean (SD)*	0.5 (1.0)	0.1 (0.3)	0.8 (1.2)	< 0.001
Nail involvement (%)				0.173
No nail involvement	72.9	82.8	65.9	
Partial nail involvement	27.1	17.2	34.1	

*Note:* Data were presented as median (interquartile range) or mean (standard deviation) for continuous variables, and as percentages for categorical variables. Wilcoxon rank‐sum test or independent *t*‐test were used for continuous variables, and Fisher's exact test for categorical variables.

Abbreviations: AA, alopecia areata; ClinRO, Clinician‐Reported Outcome; IQR, interquartile range; SALT, severity of alopecia tool; SD, standard deviation.

* *P* < 0.05.

In patients with AA, 71.4% presented in the progressive stage upon their first clinical visit, and the mean SALT score was 52.6 (SD, 38.6). At least one area of body hair is affected in 50% of patients. The mean eyebrow ClinRO score and eyelash ClinRO score were 1.0 (SD, 1.2) and 0.5 (SD, 1.0), respectively. Nail involvement occurred in 27.1% patients. Specifically, patients with severe AA exhibited greater partial body hair loss, as well as higher Clinician‐Reported Outcome (ClinRO) scores for both eyebrows and eyelashes, compared to those with mild‐to‐moderate AA (*p* < 0.05). A total of 24.3% of patients with AA presented with comorbidities, of which the most prevalent were allergic rhinitis (11.4%) and Hashimoto's thyroiditis (7.1%).

### 3.3 Lifestyle and Quality of Life

Table **3** shows the dietary habits, outdoor activities, physical exercises, and sleep conditions of patients with AA, as well as their quality of life as evaluated by DLQI.

**TABLE 3:** Dermatology Life Quality Index scores and sleep conditions of patients with alopecia areata according to disease severity.

Parameters	Total (*N* = 70)	Patients with mild‐to‐moderate AA (*N* = 29)	Patients with severe AA (*N* = 41)	*p*‐value
Dietary habits (%)				
Balanced diet	41.4	51.7	34.1	0.218
Spicy diet	37.1	37.9	36.6	> 0.999
Low‐fiber diet	27.1	24.1	29.3	0.786
High‐sugar diet	25.7	13.8	34.1	0.094
High‐fat diet	17.1	17.2	17.1	> 0.999
High‐salt diet	11.4	6.9	14.6	0.455
Average outdoor activity time on weekdays (%)				0.803
< 0.5 h	64.3	62.1	65.9	
> 0.5 h	35.7	37.9	34.1	
Average outdoor activity time on weekends and holidays (%)				0.628
< 0.5 h	50.0	44.8	53.7	
> 0.5 h	50.0	55.2	46.3	
Physical exercise (%)				0.707
Never	67.1	62.1	70.7	
1–2 times per week	27.1	31.0	24.4	
3–5 times per week	5.7	6.9	4.9	
Average bedtime (%)				0.595
Before 12:00	71.4	75.9	68.3	
After 12:00	28.6	24.1	31.7	
Average sleep duration (%)				0.614
< 7 h	64.3	69.0	61.0	
≥ 7 h	35.7	31.0	39.0	
Fatigue or lack of energy in the past 3 months (%)				0.848
Never	14.2	10.4	17.0	
Occasionally	42.9	44.8	41.5	
Often	42.9	44.8	41.5	
Sleep quality in the past 3 months (%)				> 0.999
Poor	25.7	24.1	26.8	
Fair	48.6	48.3	48.8	
Good	25.7	27.6	24.4	
Sleep disorders (%)*				0.049
No	25.7	37.9	17.1	
Yes	74.3	62.1	82.9	
Sleep disorder description (%)				
Frequent night awakenings	35.7	24.1	43.9	0.129
Non‐restorative sleep	35.7	31.0	39.0	0.614
Vivid dreams	27.1	17.2	34.1	0.173
Difficulty initiating sleep	25.0	10.7	35.0	0.026
Difficulty returning to sleep after waking	14.3	6.9	19.5	0.178
Early morning awakening	7.1	3.4	9.8	0.395
Sleep talking	5.7	0.0	9.8	0.136
Nightmares	1.4	0.0	2.4	> 0.999
Night terrors	1.4	0.0	2.4	> 0.999
DLQI scores, mean (SD)*	16.5 (6.7)	14.4 (5.3)	17.9 (7.2)	0.028

*Note:* Data were presented as mean (standard deviation) for continuous variables and as percentages for categorical variables. An independent *t*‐test was used for continuous variables, and Fisher's exact test or chi‐square test for categorical variables.

Abbreviations: AA, alopecia areata; DLQI, Dermatology Life Quality Index; SD, standard deviation.

* *P* < 0.05.

In terms of dietary habits, 41.4% of patients had a balanced diet. However, 37.1% of patients reported a preference for a spicy diet, 17.1% for a high‐fat diet, 25.7% for a high‐sugar diet, and 11.4% for a high‐salt diet. Additionally, 27.1% of patients reported a low‐fiber diet. Regarding outdoor activity, 64.3% of patients reported engaging in less than 0.5 h of outdoor activities on weekdays, and half of the participants maintained this low activity level on both weekends and holidays. Sixty‐seven point 1% of patients reported never engaging in physical exercise, with only 5.7% exercising 3–5 times per week. For sleep conditions in the past 3 months, 71.4% of patients with AA reported bedtime before midnight (12:00), while 64.3% slept less than 7 h, and 25.7% described poor sleep quality. Sleep disorders affected 74.3% of the patients with AA, with significantly higher prevalence in patients with severe AA than in patients with mild to moderate AA (*p* < 0.05).

The mean DLQI scores for all patients were 16.5 (SD, 6.7), and higher in severe AA patients than mild‐to‐moderate AA (17.9 vs. 14.4, *p* < 0.05). Compared with patients with mild‐to‐moderate AA, severe AA patients experienced significantly greater distress in interpersonal interactions with partners, relatives, and friends, as well as in participating in sports activities due to hair loss.

### 3.4 Treatment Patterns

The treatment methods received by patients with AA include topical glucocorticoids, subcutaneous injection of glucocorticoids, topical minoxidil, phototherapy, platelet‐rich plasma therapy, oral immunomodulatory drugs (e.g., compound glycyrrhizin tablets, total glucosides of paeony capsules), oral nutritional supplements (e.g., vitamin D, iron, calcium carbonate and vitamin D3 Tablets, etc.), oral antihistamines (e.g., fexofenadine, ebastine, olopatadine, etc.), systemic glucocorticoids(e.g., intramuscular injection of compound betamethasone, oral prednisone), oral Janus kinase (JAK) inhibitors (e.g., tofacitinib, baricitinib, ritlecitinib and participation in clinical trials).

From 2018 to 2024, oral JAK inhibitors were not yet widely used, but systemic therapy remained the most common treatment at our site because patients had received first‐line topical treatments at local clinics with poor efficacy or intolerance before being referred to our tertiary hospital.

Combination therapy was used for the vast majority of patients. In mild‐to‐moderate AA patients, the most commonly used therapeutic combinations were: (1) the combination of immunomodulatory drugs and nutritional supplements (62.1%); (2) the combination of Traditional Chinese Medicine and nutritional supplements (48.3%); (3) the combination of immunomodulatory drugs and antihistamines (41.4%). In severe AA patients, the most commonly used therapeutic combinations were: (1) the combination of immunomodulatory drugs and nutritional supplements (46.3%); (2) the combination of immunomodulatory drugs and antihistamines (34.1%); (3) oral JAK inhibitors combined with nutritional supplements (29.3%), rank third alongside the combination of immunomodulatory drugs and topical glucocorticoids (29.3%).

### 3.5 The Outcome of Diseases in AA Patients

**FIGURE 1:**
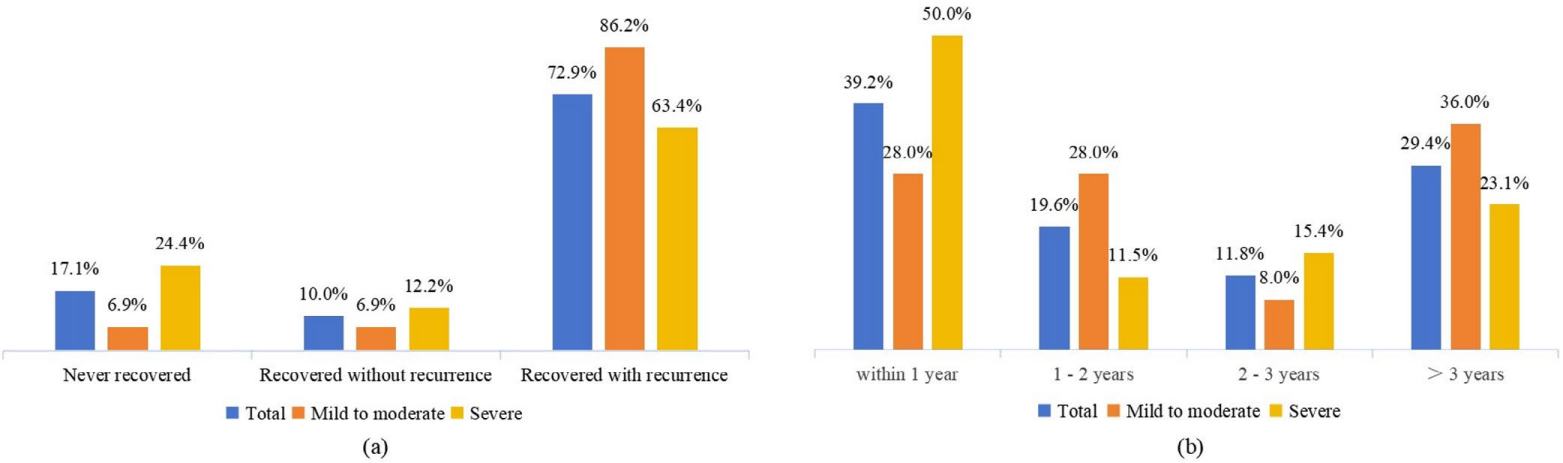
Disease course and recurrence status in patients with alopecia areata. (a) Proportions of patients with alopecia areata (AA) never achieved complete recovery (SALT = 0), recovered without recurrence, or recovered with recurrence. (b) Proportions of time to first recurrence among patients with recurrence. A total of 17.1% of patients with AA never recovered. Only 10.0% recovered without recurrence, while 72.9% of recovered patients experienced recurrence. Among patients with recurrence, the proportion of patients with recurrence within 1 year was 39.2%.

Fifty‐eight (82.9%) patients have ever recovered (SALT = 0) during the course of the disease. Fifty‐one (72.9%) patients with AA recovered with recurrence. Of these, 23 patients with AA experienced fewer than two recurrences, and 28 experienced more than two recurrences. In addition, 10.0% recovered without recurrence, while 17.1% never recovered (Figure 1a). Among patients with recurrence, the proportions of patients with recurrence within 1 year, 1–2 years, 2–3 years, and more than 3 years were 39.2%, 19.6%, 11.8%, and 29.4%, respectively (Figure 1b). Patients with mild‐to‐moderate AA were more likely to experience recurrence after three years compared to those with severe AA (36.0% vs. 23.1%). The cumulative recurrence rates were 39.2% at 1 year, 58.8% at 2 years, 70.6% at 3 years, 76.5% at 4 years, and 88.2% at 5 years. The duration of each recurrence ranged from 7.0 to 13.0 months. The time interval between recovery and recurrence varied from 7.0 to 20.0 months.

## 4 Discussion

Alopecia areata (AA) is the second most common hair loss problem in dermatological clinical practice. For both patients and doctors, the challenges of AA lie in its strong clinical heterogeneity and the unpredictability of disease outcomes, which resulted in the current lack of methods to ensure cure without recurrence. For a long time in the past, there has been a common saying in Chinese folklore that “AA can heal on its own without needing to visit a hospital.” This has caused a considerable number of AA patients to underestimate the possible progression of the disease. In this study, the median age at onset was 30.4 years, aligning with the previous studies (25.2–36.3 years) [15, 17, 18, 19]; however, the median age at diagnosis of AA was 34.4 years. Prolonged delays in obtaining timely diagnosis and therapeutic interventions not only exacerbate diseases but also undermine patients' quality of life and inflict psychological distress [20].

The evaluation of alopecia areata involves not only the extent of the hair loss area, but also classification, staging, disease course, extracutaneous involvement, and comorbidities. Patchy type AA ranges from mild single patch and multiple patches to eventual confluence, leading to total scalp hair loss. Diffuse and ophiasis types are classified mainly based on the distribution and morphology of affected lesions of alopecia areata. In this study, all the ophiasis type cases belonged to the severe AA group, which was consistent with the inherent treatment‐resistant feature of the ophiasis subtype. This finding also indicates that early diagnosis and timely intervention are essential for alopecia ophiasis, and systemic therapy should be initiated promptly according to disease progression.

In addition, disease staging is also of great importance. Most mild‐to‐moderate patients in this study were in the progressive stage at the initial visit to our site, suggesting that the condition should be evaluated dynamically with close vigilance against disease deterioration. This explains the high proportion of mild‐to‐moderate AA receiving systemic treatment in this study because many patients experienced treatment failure or intolerance to topical therapy at local clinics prior to referral to our hospital.

Although the scalp is the most commonly affected, all hair‐bearing areas can be involved in AA, and nail damage may also occur. In addition to the symptom of hair loss, 47.2%, 28.6%, 12.9%, 10.0%, and 10.0% of patients with AA presented eyebrows, eyelashes, armpit hair, beard, and pubic hair loss, respectively, and nail involvement in 27.1% of patients in this study. In this study, patients with severe AA exhibited a higher rate of body hair involvement and more severe eyebrow and eyelash scores compared with the mild‐to‐moderate group. These results were consistent with previous studies [21, 22, 23]. Nail involvement was more prevalent in the severe AA group, yet the difference lacked statistical significance.

The previous study suggested nearly 80% of patients with AA reported impaired quality of life based on DLQI survey results [24], particularly those with severe AA [25]. In our study, we also found patients with severe AA had a higher DLQI score than mild‐to‐moderate AA (17.9 vs. 14.4). Notably, the DLQI score (16.5) for all patients in our study was higher than that in previous studies (4.8–13.5) [26, 27, 28]. The discrepancy might be attributed to several factors: The high percentage of female participants in the study, the severe nature of scalp hair loss and the degree of body hair involvement, and the chronic fluctuant course of AA. These factors not only exacerbate physical symptoms but also contribute to self‐stigmatization, a phenomenon that appears to be more prevalent in AA than in other mental health conditions [29]. Therefore, the impact of AA extends far beyond an aesthetic concern, significantly affecting various aspects of patients' lives, including mental health, social relationships, romantic partnerships, and financial status [29]. Our results are consistent with the latest perspectives that the evaluation of AA should not be limited to the SALT score, but other factors should be comprehensively considered [11].

Previous studies have demonstrated that AA is associated with increased risks of atopic and other autoimmune diseases [30, 31]. In this study, 24.3% of patients with AA had comorbidities, in which allergic rhinitis and Hashimoto's thyroiditis were the most prevalent. In addition, dermatologists must not neglect inquiries about patients' lifestyles, including dietary habits, exercise, and sleep status. In this study, the incidence of sleep disturbance (insomnia) was significantly higher in severe AA patients than in mild‐to‐moderate cases. However, the correlation between insomnia and AA is complex. The onset sequence varies among individuals, and the two conditions may interact as both cause and effect, creating a vicious cycle. Further prospective studies are required for verification.

The AA treatment guidelines by different countries and regions generally formulate treatment strategies based on the severity of the condition, disease stage, and individual characteristics [11, 32, 33]. Considering the suboptimal efficacy of monotherapy, combination therapy is predominantly employed in clinical practice, particularly for the management of moderate to severe AA [34]. In this retrospective study of AA patients treated who underwent regular follow‐up for at least one year at our clinic from 2018 to 2024, 71.4% of patients presented in the progressive stage at their first visit. As the majority of patients demonstrated resistance to prior topical therapies, including topical glucocorticoids, subcutaneous glucocorticoid injections, or topical minoxidil, systemic treatments were subsequently administered. Notably, as of now, diphenylcyclopropenone, a well‐known immunotherapeutic agent elsewhere, remains unapproved for clinical use in China. Before 2023, the only systemic drug approved for the indication of AA in China was compound glycyrrhizin tablets (CGT). CGT is a glycyrrhizin‐containing (monoammonium glycyrrhizate) preparation, which has anti‐inflammatory, anti‐allergic, and immune regulatory effects [35, 36]. Other immunomodulatory drugs commonly used in the treatment of mild to moderate AA include total glucosides of paeony and hydroxychloroquine [37, 38]. In 2023, baricitinib and ritlecitinib were successively approved for the treatment of severe AA in China. In this study, the therapeutic regimen combining immunomodulatory drugs with nutritional supplements was the most frequently employed, constituting 52.9% of all patients, and JAK inhibitors were co‐administered with antihistamines in up to 19.5% of patients with severe AA. In addition, among 20 severe AA patients treated with JAK inhibitors, 11 have ever recovered (SALT = 0) for the first time.

The paramount difficulties in managing AA include treatment resistance, unpredictable recurrence, and the need to balance therapeutic safety with patients' acceptability. These factors collectively present significant challenges in clinical practice, notwithstanding substantial advancements in novel small‐molecule drugs and precision‐targeted therapeutics.

Most patients with AA (82.9%) recovered in our study, which was almost equal to the 80% reported in previous studies [39]. The vast majority of Chinese patients take complete hair regrowth (SALT = 0) as the criterion for the success of treatment. However, the concept of treat‐to‐target (e.g., SALT < 20) has been introduced into the treatment of AA [40].

While treat‐to‐target paradigms and the JAK inhibitors ameliorate clinical outcomes, sustained drug‐free remission or even cure remains elusive. Although approximately 50% of patients with AA recover within one year after the first episode [14], a report has indicated that all AA patients will experience recurrence if followed up for a sufficiently long period [41]. This finding underscores the necessity of long‐term maintenance treatment. Our study demonstrated a post‐recovery recurrence rate of 72.9%, which is significantly higher than the 44.0% recurrence rate previously reported in previous studies [39]. This notable discrepancy may be attributed to differences in patient demographics, a higher percentage of severe AA, treatment protocols, or follow‐up durations employed across studies. The cumulative recurrence rates in our study further emphasized the importance of long‐term treatment. The time between recovery and relapse varied from 7.0 to 20.0 months, with each recurrence lasting 7.0 to 13.0 months. These variations may arise from interindividual variability in immune responses, treatment regimens, and disease progression. Regarding recurrence frequency, 23 patients experienced fewer than two relapses, while 28 experienced more than two. Variability in treatment response and recurrence rates complicates the selection of optimal therapy for AA. A previous study has pointed out the uncertainty surrounding therapeutic decisions and highlighted the need for personalized treatment plans [42]. Additionally, patients with mild‐to‐moderate AA tended to relapse later than those with severe AA, with 36.0% of mild‐to‐moderate patients relapsing after three years, compared to 23.1% of severe AA patients. This may be due to more aggressive disease in severe AA, which leads to earlier relapses.

Limitations of this study include the retrospective nature of the data, small sample size, and lack of multi‐center investigation. In addition, the time frame of this retrospective survey was from 2018 to 2024. Therefore, a considerable number of patients did not receive JAK inhibitor treatment at that time due to the lack of approved indications in China or unaffordable treatment costs (In general, newly launched drugs tend to experience a price reduction approximately 3–5 years after being launched on the market in China.), which led to the result that the treatment patterns could not exactly match the current situation. Further prospective multicenter cohort studies with a large sample size should be conducted to verify the results of this study.

## 5 Conclusion

In conclusion, this retrospective study described the clinical characteristics, quality of life, treatment patterns, and long‐term prognostic outcomes in Chinese patients with AA. Current treatments have yet to achieve optimal outcomes in complete hair regrowth and reduction of recurrence, and there remain significant unmet needs in the treatment of AA. For moderate to severe AA, a treatment‐to‐target strategy with remission as the therapeutic goal should be emphasized, and more personalized treatment approaches are advocated. Under this strategy, when prior treatment proves ineffective, systemic therapy targeting the underlying pathophysiological mechanisms of the disease should be initiated promptly.

## Author Contributions

All authors have read and approved the final manuscript. All authors contributed to the interpretation of data and critical revision of the manuscript. Y.S., Z.Q., J.Z., L.Z., and Y.M. performed the research. W.W., Q.P., and G.D. designed the research study. B.Z. and J.L. contributed essential reagents or tools. Y.S., Z.Q., and J.Z. analyzed the data. Y.S., Z.Q., and J.Z. wrote the paper.

## Funding

This study was financially sponsored by Eli Lilly (Shanghai, China) under internal project ID 2021‐11042, yet the sponsor played no role in data collection or data analysis throughout the research.

## Ethics Statement

The authors confirm that they have adhered to the ethical policies of the journal and that this study obtained approval from the Institutional Review Board of Huashan Hospital, Fudan University (Approval No. 2021–11 042). The study was conducted in accordance with the ethical standards of the institutional research committee and the 1964 Helsinki Declaration and its later amendments. Written informed consent was obtained from all individual participants prior to their inclusion in the study.

## Conflicts of Interest

Youyu Sheng is a clinical trial investigator for Pfizer, Eli Lilly, AbbVie, Sanofi, and Reistone Biopharma. Jun Zhao is a clinical trial investigator for Pfizer and Eli Lilly. Ying Miao is a clinical trial investigator for Pfizer, Eli Lilly, AbbVie, and Reistone Biopharma. Wenyu Wu is a clinical trial investigator for Pfizer, Eli Lilly, AbbVie, and Sanofi. Qinping Yang is a clinical trial investigator for Pfizer, Eli Lilly, and Reistone Biopharma. Zhibing Qiu, Lijuan Zhou, Bilian Zhao, Jinnan Li, and Guanshen Dou declare no conflicts of interest.

## Data Availability

The data that support the findings of this study are available on request from the corresponding author. The data are not publicly available due to privacy or ethical restrictions.
